# Diaphragmatic Ultrasound Advantages in Chronic Obstructive Pulmonary Disease (COPD) Patients: A Systematic Review and Metaanalysis

**DOI:** 10.4314/ejhs.v33i5.20

**Published:** 2023-09

**Authors:** Hadi Eshaghi Sani Kakhaki, Samira Alesaeidi, Goli Siri, Amir Arya, Hadi Sarafraz, Khadijeh Otadi, Niloofar Ayoobi Yazdi, Kobra Abedinzadeh

**Affiliations:** 1 Department of Occupational Medicine, School of Medicine, Hormozgan University of Medical Sciences, Bandar Abbas, Iran; 2 Department of Internal Medicine, School of Medicine, Tehran University of Medical Sciences, Tehran, Iran; 3 Department of Physiotherapy, Petroleum Industry Health Organization, Tehran, Iran; 4 Department of Physiotherapy, School of Rehabilitation, Tehran University of Medical Sciences, Tehran, Iran; 5 Department of Radiology, School of Medicine, Advanced Diagnostic and Interventional Radiology Research Center, Imam Khomeini Hospital Complex, Tehran University of Medical Sciences, Tehran, Iran; 6 Department of Emergency Medicine, Persian Gulf Hospital, Bandar Abbas, Iran

**Keywords:** Chronic Obstructive Pulmonary Disease, diaphragm ultrasound, FEV1, FVC, systematic review, meta-analysis

## Abstract

**Background:**

Diaphragmatic ultrasound is increasingly used to assess patients with Chronic Obstructive Pulmonary Disease (COPD). The present study aims to investigate diaphragmatic dysfunction in COPD patients through a systematic review and meta-analysis.

**Methods:**

In December 2022,The researchers studied four international databases such as Medline/PubMed, ProQuest, ISI/WOS, and Scopus. Joanna Briggs Institute (JBI) checklist was used to review and control the quality of articles.

**Results:**

Finally, 6 articles were included in the analysis. Based on the meta-analysis results, forced expiratory volume (FEV1) was significantly lower in COPD patients compared to the control group (Hedges's g= -2.99, 95 % CI -4.78, -1.19; P =0.001). Forced vital capacity (FVC) was significantly lower in COPD patients compared to the control group (Hedges's g= -1.12, 95 % CI -1.91, - 0.33; P =0.005). COPD patients had significantly lower FEV1/FVC than the control group (Hedges's g= -1.57, 95 % CI -2.33, -0.81; P <0.001).

**Conclusion:**

The present study showed that the diaphragm ultrasound (DUS) method could identify the difference in FEV1, FVC, and FEV1/FVC indices in two groups of COPD patients and healthy people.

## Introduction

Chronic obstructive pulmonary disease (COPD) is the fourth leading cause of death worldwide, and its incidence and burden will increase in the following years (1). Since the past decades, different comorbidities and non-pulmonary complications of COPD have attracted the attention of researchers worldwide (2). Previous studies evaluated this aspect of COPD and addressed this disease as a multifaceted entity (2-4). Occupational factors play an essential role in the progress of COPD. Recent evidence shows a causal association between multiple categories of occupational exposure and COPD (5-7).

This disease is characterized by progressive obstruction of the airways, which is irreversible to some extent (8). Diaphragm function is an essential factor in the pathophysiology of this disease. The mechanism of dyspnea in COPD includes increased workload by the respiratory muscle system and a systemic neuromechanical coupling, which is indicated by the electrical activities of the diaphragm (9). Peripheral muscle dysfunction, weakness, and sarcopenia are recognized as complications of COPD and are associated with disease severity (10).

In patients with COPD, the increased airway resistance and airflow restriction increase the mechanical load and work of breathing through the diaphragm. Dynamic hyperinflation also impairs diaphragmatic function by shortening the diaphragm to less than optimal length, reducing the curvature of the diaphragm, and reducing the contact area of the diaphragm with the chest wall. These mechanisms place the diaphragm at a mechanical disadvantage, increasing its workload and reducing power output (11).

Diaphragm assessment is essential for COPD patients but challenging to achieve. The gold standard for evaluating diaphragm function is measuring transdiaphragmatic pressure using an electromyogram during phrenic nerve stimulation or through maximal static inspiratory pressure (12). However, its use is minimal because it is an invasive and time-consuming technique. These techniques have limitations, including radiation exposure, the need for invasive procedures and technical challenges (13,14).

Ultrasonography can effectively evaluate diaphragm dysfunction in COPD patients (15). Diaphragm ultrasound (DUS) is an emerging alternative method for assessing the diaphragm muscle. Ultrasound provides a real-time, noninvasive method for evaluating the diaphragm and is increasingly used in clinical practice. Ultrasound can determine muscle structure (diaphragm thickness), mobility (diaphragm movement), activity (thickness fraction), and function (maximal thickening fraction) of the diaphragm (16,17). This technique and its reproducibility have been widely evaluated in healthy subjects (18,19), outpatients (20), and critically ill patients (21). Ultrasound may facilitate assessment and monitoring diaphragmatic dysfunction in patients with COPD. However, to date, such data in patients with COPD are limited.

Previous studies discussed the role of DUS as an imaging marker for COPD patients. Catherine et al. used this method in a prospective cohort study. They stated that in patients with COPD, ultrasound evaluation of the quadriceps contraction index is possible and is related to the severity of the disease, clinical symptoms, history of exacerbations, and diaphragm contraction. However, further studies are needed to determine better its potential role as a prognostic marker in this population (22,23).

Okura et al. also showed that diaphragm ultrasound could evaluate diaphragm dysfunction related to COPD, but the results need further investigation (24). So far, various studies have been conducted that have had different results and have stated that more studies are required to confirm this method's effectiveness on COPD. One of the ways to summarize the results of published studies and provide a unified result is to conduct systematic review and meta-analysis studies. Hence, the present study aims to investigate the effectiveness of the DUS method for COPD patients through a systematic review and meta-analysis.

## Methods

**Setting**: The present study is a systematic review and meta-analysis of the effectiveness of the DUS method as an imaging marker for COPD patients. The study was designed and conducted in 2022. The present study's reporting method was based on the PRISMA (Preferred Reporting Items for Systematic Reviews and Meta-Analysis) checklist (25).

**Search strategy**: In December 2022, the researchers studied four international databases: Medline/PubMed, ProQuest, Web of Since, and Scopus. The selected keywords for databases included “diaphragm ultrasound” OR “ultrasonography” OR “ultrasound evaluation of diaphragm” AND “Chronic obstructive pulmonary disease” OR “COPD”. The collected articles were entered into EndNote, X8 software, and duplicate reports were automatically deleted. The two researchers examined the papers separately.

**Inclusion criteria**: Based on its purpose, the present study included only studies conducted on the effectiveness of the DUS method as an imaging marker for COPD patients compared to healthy subjects.

**Exclusion criteria**: The articles have these criteria were excluded: without full text, only in COPD patients without a control group, and also received a qualitative assessment score of less than 4.

**Quality assessment**: Joanna Briggs Institute (JBI) checklist was used to review and control the quality of articles. This checklist is used for a wide range of studies (26).

**Screening of studies**: The initial search was conducted by two researchers (X and Y). Screening of studies, extraction of results, and evaluation of quality of articles were performed separately by two researchers (A and B). If there were no agreements between the two, the team leader (C) would announce the final opinion on that article.

**Statistical analysis**: The heterogeneity of the studies was investigated by Cochran's test (with a significance level of less than 0.1) and its combination using I^2^ statistics (with a significance level greater than 50%). In the case of model heterogeneity, random effects were used by the variance image method. The standardized mean difference (SDM) and Hedges's g were used to combine the results of different studies. The Hedges' g index provided the ability to combine studies that reported results in different ways. Due to the high heterogeneity in the metaanalysis results, a power analysis was used to estimate the power of effect sizes. All analyzes were performed by CMA statistical software version 2.

## Results

**Figure 1:**
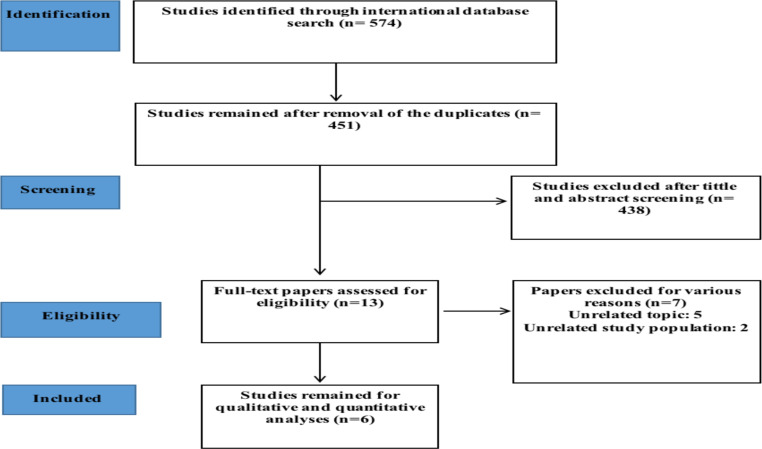
Flowchart of the included eligible studies in the systematic review

**Description of searched studies**: After searching all the databases, 574 articles were found, and after deleting duplicate articles, 451 articles entered the review stage in terms of title and abstract. In total, 13 studies met the inclusion criteria and entered the second evaluation stage. After several screening phases, 6 articles were included in the final analysis. The references to the submitted articles were also reviewed to add relevant studies. In the screening stages, the studies were excluded from the study for a variety of reasons, including unrelated topics (378), unrelated study populations (66), and repetitive results (1). The flowchart of the studies is shown in Figure 1.

**Description of the included studies**: The characteristics of the included studies (27-32) are presented in Table 1, based on the geographical location of the 6 included studies: one conducted in Brazil, one in Thailand, one in Japan, one in Korea, one in the Netherlands, and one in Canada. Design-wise, 3 were cross-sectional studies, and 3 were cohort studies. The sample size range in the studies was between 13 and 100 cases.

**The results of the quality assessment**: Three studies were of high quality, and three were of medium quality.

## Results

**Figure 2:**
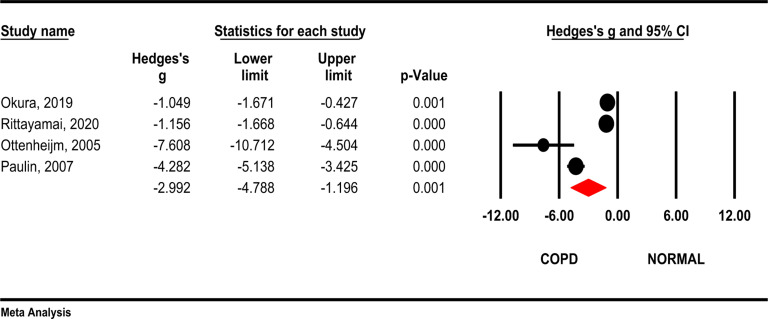
Comparison of FEV1 between COPD patients and healthy subjects

**Forced Expiratory Volume (FEV1)**: Based on the results of the random effect meta-analysis, the forced expiratory volume (FEV1) was significantly lower in COPD patients compared to the control group (Hedges's g= -2.99, 95 % CI - 4.78, -1.19; P =0.001; I2= 94.7 %, *P*<0.001). (Figure 2).

**Figure 3:**
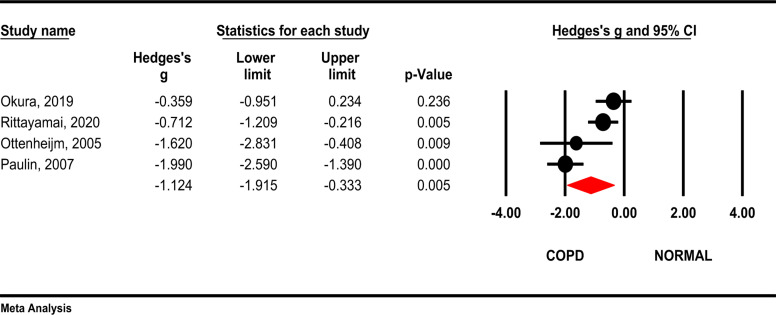
Comparison of FVC between COPD patients and healthy subjects

***Forced Vital Capacity (FVC):*** Results of the meta-analysis showed that the forced vital capacity (FVC) ratio of FEV1 and FVC was significantly lower in COPD patients compared to the control group (Hedges's g= -1.12, 95 % CI - 1.91, -0.33; P =0.005; 12= 82.5 %, *P*<0.001) (Figure 3).

**Figure 4:**
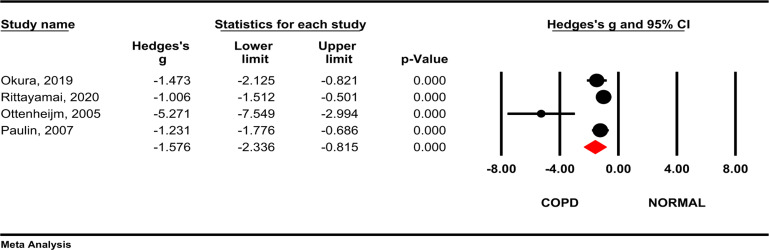
Comparison of FEV1/FVC between COPD patients and healthy subjects

**FEV1/FVC**: Results of our study showed that COPD patients had significantly lower FEV1/FVC than the control group (Hedges's g= - 1.57, 95 % CI -2.33, -0.81; *P* <0.001; I2= 77.4 %, *P*=0.004) (Figure 4).

**Figure 5:**
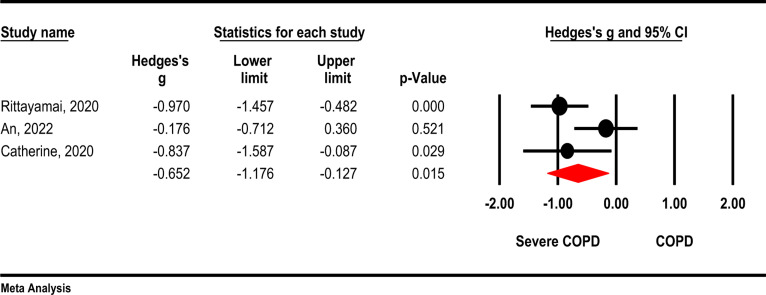
Comparison of FEV1/FVC between severe COPD patients and normal COPD

**TFdi-tidal**: Based on the results of random effect meta-analysis, TFdi-tidal was significantly lower in severe COPD patients than in normal COPDs (Hedges's g= -0.65, 95 % CI -1.17, -0.12; *P* =0.015; I2= 59.1 %, *P*=0.086). (Figure 5).

**Power analysis**: The results of the power analysis are presented in Table 2. According to the power analysis results, the meta-analysis results were unaffected by heterogeneity.

**Table 1:** The primary characteristic of included studies

Author, year	Country	Design	Sample size	Age	Population	Quality score
Rittayamai, 2020	Thailand	a prospective controlled cohort study	80/20	Median: 71 years (COPD), 72 years (Control)	Patients with stable COPD (n = 80) and healthy control subjects (n = 20)	High
Paulin, 2007	Brazil	prospective cohort	54/20	Mean: 62.1 years (COPD), 58.3 years (Control)	Fifty-four COPD patients and twenty healthy (age- and body mass index-matched) controls	Moderate
Ottenheijm, 2005	Netherlands	cross-sectional	8/5	Mean: 60 years (COPD), 59 years (Control)	eight patients with COPD (six men) and five patients without COPD (three men)	Moderate
Okura, 2020	Japan	cross-sectional	38/15/15	Mean: 72 years (COPD), 72 years (Old control), 22 years young control	Thirty-eight male patients with COPD, 15 healthy younger, and 15 healthy older male volunteers	High
Catherine, 2020	Canada	prospective cohort	31/9	Mean: 66	COPD patients (31 COPD and nine severe COPD patients)	Moderate
An, 2022	Korea	cross-sectional	33/22	Mean: 73.5 years stable COPD and 72.6 severe COPD	33 Stable COPD and 22 severe COPD	High

**Table 2:** Result of power analysis

Index	I^2^ (%)	Level of heterogeneity	(1-β error probability)
FEV1	94.7	High heterogeneity	1.0
FVC	82.5	High heterogeneity	1.0
FEV1/FVC	77.4	High heterogeneity	1.0
TFdi-tidal	59.1	Medium heterogeneity	0.94

## Discussion

The present study was conducted to investigate the effectiveness of the DUS method as an imaging marker for COPD patients and review the results of previous studies. The results showed a significant relationship between the pulmonary function test with the forced expiratory volume (FEV1), forced vital capacity (FVC), and the ratio of FEV1 and FVC in the two groups of patient and control. Also, Tfdi tidal index significantly differed in severe COPD compared to non-severe COPD. These findings show the applicability of the DUS indicator in identifying COPD. The results of the present meta-analysis based on four studies showed significant differences between the two groups of COPD patients and healthy subjects regarding FEV1, FVC, and FEV1/FVC using the DUS method.

Lung diseases can be seen in two forms: obstructive and restrictive. Restrictive diseases (such as pulmonary fibrosis) affect a person's ability to inhale, and obstructive conditions (such as asthma and COPD) affect a person's ability to exhale (33). Several indicators are reported in spirometry tests to identify these two states. The three leading indicators are FEV1, FVC, and FEV1/FVC. FEV1 means the forced expiratory volume in the first 1 second of breathing, which indicates weakness in the person's respiratory system. FVC is the total amount of air that can be exhaled with effort in a full breath, and FEV1/FVC is a ratio which shows the amount of air that a person can forcefully exhale from his lungs. According to studies, these indices are three accepted indicators of COPD disease (34). In addition, some studies divide COPD patients into four groups based on these indicators, patients with FEV1 value over 80% as mild COPD, between 80% and 50% as moderate COPD, between 49% and 30% as severe COPD, and <29% as having very severe COPD (35). Therefore, detecting the changes of these three indicators in one method can indicate the method's effectiveness in diagnosing the disease. Based on the results of the present study, we concluded that the DUS method, with the ability to detect these three indicators in COPD patients, is a suitable method for diagnosing the disease compared to healthy people.

Previous studies showed that decreased diaphragm mobility is associated with reduced physical and ventilator capacities and increased shortness of breath during exercise in COPD patients. The loss of diaphragm mobility detected by DUS in this study is similar to previous studies (8,36). Also, according to Laplace's law, which is widely accepted, pulmonary inflation leads to mechanical damage to the diaphragm. The reviewed studies showed that the reduced mobility of the diaphragm in COPD patients occurs mainly due to air trapping and is unaffected by pulmonary inflation. Clinically, decreased diaphragmatic mobility is related to the volume of air the patient can exhale, not the volume that the patient can inhale (37). Therefore, detecting FEV1, FVC, and FEV1/FVC indicators is more important, and the DUS method was able to show their difference in the two groups.

Determination of FEV1, FVC, and FEV1/FVC is essential to diagnose and quantify COPD-related impairment. However, it does not reflect the systemic manifestations of the disease or the patient's functional impairment. Recent studies have questioned the use of these indices alone as an outcome measure for various interventions or as a measure of disease severity in patients with COPD (8,38). Therefore, the present study investigated another index called TFdi tidal in two groups of severe and regular COPD patients. This index is the maximal diaphragmatic thickening fraction (TFdi-max), which measures diaphragmatic performance during maximal isometric inspiratory effort. This tidal index is higher during resting breathing in patients with COPD than in controls, indicating higher tidal diaphragmatic activity. Patients with COPD also have poorer diaphragm function and TFdi-tidal detection compared to control subjects (39). In this condition, the diaphragm must create more force than expected to move air into the lungs (40). Also, as previous studies have shown, TFdi-tidal in patients with severe COPD (FEV1 less than 50%) is significantly lower than in control subjects (13), which the DUS method also showed in the previous studies.

In conclusion, the present study showed that the DUS method could identify the difference in FEV1, FVC, and FEV1/FVC indices in two groups of COPD patients and healthy people. Also, the DUS method could recognize TFdi-tidal between severe and normal COPD patients, showing a significant difference. Therefore, it can be concluded that the DUS method as an imaging marker and non-destructive method can diagnose COPD.

The present study has limitations, including the few studies using the DUS method to diagnose COPD. We suggest further studies using this method for evaluating COPD patients. The present study showed a significant relationship between the pulmonary function test with the FEV1, FVC, and the ratio of FEV1 and FVC in the two groups of patient and control. It can be suggested that the DUS is a suitable indicator for identifying COPD, and it is suggested that future studies use this method to identify COPD patients.
